# Lima LV, Pavinati G, Silva RGT, Queiroz IG, Oliveira GLA, Durães SMB, López MAA, Alves KBA, Magalhães FB, Magnabosco GT. Leprosy elimination phase in Alagoas, Brazil, 2001-2022: an ecological study. Epidemiol Serv Saude. 2025;34:e20240255. Doi: 10.1590/S2237-96222024v34e20240255.en

**DOI:** 10.1590/S2237-96222024v34e20240255.b

**Published:** 2025-04-11

**Authors:** Maria Luiza Ferreira Imburana da Silva

**Affiliations:** 1 Universidade Federal de Pernambuco, Recife, PE, Brasil

## Peer review B

## Questionnaire

Does the manuscript contain new and significant information to justify publication? Yes

Does the Abstract (Summary) clearly and accurately describe the content of the article? Yes

Is the problem significant and concisely stated? Yes

Are the methods described comprehensively? Yes

Are the interpretations and conclusions justified by the results? Yes

Is adequate reference made to other work in the field? Yes

## Manuscript Structure

Length of article is: Adequate

Number of tables is: Adequate

Number of figures is: Adequate

Please state any conflict(s) of interest that you have in relation to the review of this paper (state “none” if this is not applicable).

None

Interest: Excellent

Quality: Good

Originality: Good

Overall: Good

Recommendation: Major Revision

## Comments

O texto aborda doença de importância para área da saúde , principalmente por ser uma das doenças mais negligenciadas. 

A discussão aponta características sociodemográficas que poderiam ser aprofundada com menção das particularidades do local do estudo. 

Nos municípios com maiores taxas médias de prevalência, taxa de detecção na população geral e < 15 anos. 

Quais são as semelhanças (sociais, demográficas, econômicas, culturais, étnicas, históricas) e as principais diferenças? Além disso, explorar as possíveis causas dessas diferenças, utilizando dados da literatura para fortalecer essas posições. 

O estudo cita a ausência de casos em determinados municípios alagoanos. Quais são esses municípios? 

Essas informações são imprescindíveis para que seja possível levantar algumas hipóteses para estudos futuros. 

Na conclusão explique as implicações práticas das descobertas, discutindo sua relevância.

